# Do Nostrums Benefit Consumers?

**Published:** 1879-11

**Authors:** 


### DO NOSTRUMS BENEFIT CONSUMERS?

In practical medicine the principle is well established that a remedy may prove beneficial at one stage of a disease, while in another, serious injury may result. Should physicians themselves think proper to use secret preparations, they could not do so safely to their patients without knowing all the ingredients entering into them. Certain useful articles of medicines cannot be used without great detriment under certain circumstances in diseases, though under other circumstances they prove highly useful. For example, tartar emetic is a potent remedy as a nauseant and sedative in acute pulmonary and bronchial affections when no circumstance contra-indicates its use. If, however, gastric or enteric irritation or inflammation complicates the case, fatal termination may result from the increase of inflammation in the alimentary mucous membrane by this local irritant. It is said to be an ingredient in certain nostrums, Ayer's Cherry Pectoral, for instance, and any of them which contain it are capable of much harm, particularly in the hands of those who are not only ignorant of the presence of this irritant, but of its action also. The same may be said of various other powerful remedies entering into the preparation of nostrums, which are freely used by the people without even the advice of a physician being asked.

Manufacturers and dealers study human nature instead of the art of healing, and by ingenious advertisements and certificates, place before the eyes of invalids that which will tempt them from confidence in their consciencious, faithful physician, and the money which should go toward the payment of his fees for useful advice and laborious care, is thrown away upon the cormorant who seeks his own gain, ignorant and regardless of the welfare of his victim.

We unhesitatingly declare the opinion, that nostrums not only afford no benefit to the people, but are injurious physically and pecuniarily. It is not alone from selfish motives that physicians denounce secret preparations, but as humanitarians and honest neighbors they advise against this evil.

Medical men often withhold their advice and caution on account of the general feeling which exists among the people that physicians oppose nostrums only because their business is injured by them. The interest of dealers is promoted by encouraging this feeling in the community, and it is likely they do so.

The great advantage nostrum venders have over physicians is that while with circulars, certificates, posters, and newspaper advertisements, the former keep their nostrums before the people, the latter make no blast of trumpets, but depend for success upon their medical knowledge and also the good sense of the people, which is often warped by ingenious charlatans.

